# StudiCare procrastination - Randomized controlled non-inferiority trial of a persuasive design-optimized internet- and mobile-based intervention with digital coach targeting procrastination in college students

**DOI:** 10.1186/s40359-023-01312-1

**Published:** 2023-09-12

**Authors:** Agnes Mutter, A.-M. Küchler, A. R. Idrees, F. Kählke, Y. Terhorst, H. Baumeister

**Affiliations:** 1 Department of Clinical Psychology and Psychotherapy, Institute of Psychology and Education, Faculty of Engineering, Ulm University, Ulm, Germany; 2 Institute of Databases and Information Systems (DBIS), Ulm University, Ulm, Germany; 3 Department of Sport and Health Sciences, Professorship of Psychology and Digital Mental Health Care, Technische Universität München, Munich, Germany

**Keywords:** Persuasive System Design, Internet- and mobile-based cognitive behavioral therapy, Procrastination, Digital guidance

## Abstract

**Background:**

Academic procrastination is widespread among college students. Procrastination is strongly negatively correlated with psychological well-being, thus early interventions are needed. Internet- and mobile-based cognitive behavioral therapy (iCBT) could provide a low-threshold treatment option. Human guidance seems to be a decisive mechanism of change in iCBT. Persuasive design optimization of iCBT and guidance by a digital coach might represent a resource-saving alternative. The study evaluated the non-inferiority of a digital coach in comparison to human guidance with regard to the primary outcome procrastination.

**Methods:**

The iCBT StudiCare procrastination was optimized by principles of the Persuasive System Design (PSD). A total of 233 college students were randomly assigned to either StudiCare procrastination guided by a digital coach (intervention group, IG) or by a human eCoach (control group, CG). All participants were assessed at baseline, 4-, 8- and 12-weeks post-randomization. Symptom change and between-group differences were assessed with latent growth curve models and supported by effect size levels. The non-inferiority margin was set at Cohen’s *d* = − 0.3.

**Results:**

The primary outcome procrastination measured by the Irrational Procrastination scale (IPS) significantly decreased across groups (γ = − 0.79, *p* < .001, Cohen’s *d* = -0.43 to -0.89) from baseline to 12-weeks post-randomization. There were no significant differences between groups (γ = -0.03, *p* = .84, Cohen’s *d* = -0.03 to 0.08). Regarding symptoms of depression, no significant time x group effect was found (γ = 0.26, *p* = .09; Cohen’s *d* = -0.15 to 0.21). There was also no significant time x group effect on the improvement of symptoms of anxiety (γ = 0.25, *p* = .09). However, Cohen’s *d*s were above the non-inferiority margin 8-weeks (Cohen’s *d* = 0.51) and 12-weeks post-randomization (Cohen’s *d* = 0.37), preferring the CG. Of the IG, 34% and of the CG, 36% completed 80% of the modules.

**Conclusions:**

The PSD optimized version of StudiCare procrastination is effective in reducing procrastination. The digital coach was not inferior to human guidance. Guidance by a digital coach in iCBT against procrastination for college students could be a resource-saving alternative to human guidance.

**Trial registration:**

The trial was registered at the WHO International Clinical Trials Registry Platform via the German Clinical Trial Register (ID: DRKS00025209, 30/04/2021).

**Supplementary Information:**

The online version contains supplementary material available at 10.1186/s40359-023-01312-1.

## Background

Academic procrastination, the phenomenon of postponing intended and necessary study-related tasks despite the expectation of negative consequences, is widespread among college students [1]. In the general population studies report procrastination prevalence rates of 15–46% [2, 3]. However, depending on the definition and assessment tool applied, some research has reported prevalence rates for academic procrastination of up to 95% [4–7]. Yan et al. [8] summarized the current literature on procrastination in a systematical bibliometric analysis. Research suggests, procrastination to be a behavioral tendency, which is complex and stable across different situations. From a differential psychology perspective procrastination is seen as a personality trait [1]. In the motivational and volitional psychology it is recognized as a self-regulation failure [1, 9] including behavior and emotion associated with situational and personal determinants [10]. Cognitive and motivational process, personality traits (e.g., impulsivity [11]), and contextual conditions (e.g., task characteristics [12]) are profound reasons for procrastination [8]. Among other negative effects, procrastination is highly correlated to increased levels of stress [13], symptoms of depression and anxiety [14], poor academic performance [15], and poor general health [16]. Cognitive behavioral therapy (CBT) may be an effective treatment to reduce procrastination in students in the short- and the long-term [17]. Due to the complexity, high prevalence and negative outcomes of procrastination, it could be beneficial to provide students with low-threshold access to CBT. To do so, CBT can be delivered via internet- and mobile-based interventions (IMI). For students, IMI may be appealing, given their flexibility and anonymity [18]. Studies on IMI targeting procrastination report promising results in reducing procrastination and improving symptoms of anxiety and depression [19–21]. Rozental et al. [19] found a reduction of procrastination with an effect of Hedges’ *g* = 0.69 (95%-CI: 0.28; 1.09) of a human guided iCBT compared to a wait-list control condition. The Internet- and mobile-based cognitive behavioral therapy (iCBT) StudiCare procrastination, which was investigated in this study, is also currently being evaluated in reducing procrastination in a guided intervention group compared to a waiting-list control group [22, 23].

Research suggests guidance to be a decisive mechanism of change in IMI, likely due to its motivation and engagement facilitating effect [24, 25]. A meta-analysis including individual participant data of patients with depression reveals that the combination of human and automated support decreases IMI’s dropout rates [26]. However, since human guidance is resource intensive, it may be beneficial to find alternative motivation- and engagement-facilitating strategies to make IMI even more accessible and scalable, e.g., by implementing Persuasive System Design strategies (PSD) [27]. By the effective use of human-machine interaction, PSD, in the form of information systems, pursue the goal of achieving behavioral or attitudinal change in individuals [27]. Previous studies report on the positive influence of PSD in IMI in terms of motivating behavior change and influencing attitudes of users and thereby potentially increasing user adherence [28, 29]. Dialogue support, one of four subcategories of PSD, portrays the machine in a social role, e.g., by implementing an avatar-based coach leading through the IMI. The qualifications of the guiding coach do not play a significant role in the positive effect of human guidance in IMI [24]. Hence, the question arises, whether a digital avatar-based coach might produce similarly effective results as a human coach. Previous studies indicate that other forms of automated support (e.g., by email support) can be effective but less so than human guidance [30]. PSD was found to be a resource-saving solution to improve adherence [28]. The PSD optimization might also reduce the inferiority of automated guidance. Research suggests that it is possible to establish a working alliance with an avatar and that this alliance might predict symptom change [31]. A scoping review demonstrates that embodied conversational agents (e.g., avatars) can be used for the treatment of various mental illnesses [32]. This review includes some studies in which this agent was implemented as an adjunct to a self-management intervention. The results of included evaluation phase studies point to a potentially positive effect of the agents in terms of user engagement. It should be noted that there is currently a lack of component studies in research that directly compare the guidance of a digital coach to human guidance [32]. One of the few existing component studies is from Kelders et al. (2015) [33]. In a pilot study an IMI targeting people with depression, no difference in adherence between the group accompanied by automated feedback compared to the group with human guidance was found.

The combination of automated support with further PSD principles may be a resource-saving alternative to guidance in IMI to increase users’ motivation and engagement and thus might be equally effective as human guidance.

Since the existing research in this field is still limited, this study aims to make an important contribution based on the following research questions:

1. Is the guidance by a digital coach non-inferior to the active control group guided by a human coach regarding the reduction of procrastination (primary outcome)?
2. Is the guidance by a digital coach non-inferior to the active control group guided by a human coach regarding the improvement of students’ depression, anxiety, susceptibility to temptation, self-efficacy, perceived stress, and social support?
3. Does StudiCare procrastination improve procrastination, depression, anxiety, susceptibility to temptation, self-efficacy, perceived stress, and social support in college students?
4. Are there potential predictors of the effect across groups on procrastination, depression, anxiety, susceptibility to temptation, self-efficacy, perceived stress, and social support?
5. Is StudiCare procrastination with digital coach non-inferior to the active control group guided by a human coach in terms of feasibility, as measured by therapeutic alliance, adherence, usability, dropout, and reported subjective negative side effects?

## Materials and methods

This parallel, two-group, randomized controlled trial evaluated the non-inferiority of a PSD optimized iCBT guided by a digital coach (IG) or a human coach (CG). This trial was a-priori registered at the WHO International Clinical Trials Registry (ID: DRKS00025209; 30/04/2021), approved by the ethic committee of Ulm University (Number 502/20) and was conducted and reported according to the extension of the CONSORT 2010 Statement for non-inferiority and equivalence trials [34, 35].

### Participants and inclusion criteria

Participants were eligible for participation when meeting the following requirements: (1) self-reported age of 18 years and older, (2) sufficient German language skills, (3) self-reported enrollment in university or college, (4) internet access, (5) ability to use a smartphone. As there is no clinical diagnosis of procrastination and in order to include students significantly suffering from it a cut-off value based on a recent study [19] was applied (Irrational Procrastination Scale (IPS) ≥ 32). There were no exclusion criteria. Participants were recruited via a website, postings to Facebook, flyers at Ulm University and circular emails with study information sent out by the University of Hannover, Siegen, and Rostock between May and September 2021 in Germany.

Interested students were invited to conduct an eligibility screening on the StudiCare website. In case of eligibility, participants had to give their informed consent online via double opt-in. Subsequently, they were invited to the baseline assessment.

### Randomization and sample size

We performed an a-priori sample size calculation based on a power of 0.90 and α = 0.05. We assumed a non-inferiority margin of Cohen’s *d* = -0.3, and a standard deviation (*SD*) of 0.78 [36] based on the severity of procrastination as primary outcome. The selection of the margin was based on clinical judgement and statistical reasoning [37]. We followed the recommendation that the non-inferiority margin should not be greater than the smallest effect expected for the control condition in a superiority RCT [37]. Thereby, we related to the existing studies targeting procrastination with iCBT [19, 38]. We noted that the authors concluded that clinical significance in procrastination needs to be investigated further, and we aligned with their proposed relevant standard deviations. Furthermore, we researched on commonly used non-inferiority margins [39] as well as currently available approaches to defining clinically significant effects in psychological research [40, 41]. The calculation resulted in *N* = 232 participants.

Participants were randomly assigned to IG or CG in a 1:1 ratio with a permuted block size (4,6,8) by a researcher not otherwise involved in the study using a web-based automated randomization program (https://www.sealedenvelope.com). Study staff concerned with outcome assessment were blinded to participants’ group allocation. Participants were informed about their group assignment via email.

### Intervention procedure

StudiCare procrastination is a self-help iCBT [42] developed by Ulm University Department of Clinical Psychology and Psychotherapy. Content is presented through interactive elements as audio, video, illustrations, and text enriched by writing-based or multiple-choice tasks. The intervention consists of an introduction, five required modules, and one optional module. Participants in the current study were encouraged to complete modules on a weekly basis. Modules target (1) psychoeducation about procrastination; (2) time-management strategies and goal-setting; (3) motivation; (4) self-regulation and mindfulness; (5) relapse prevention. Detailed information on content of each module can be found elsewhere [22]. Since studies have also found correlations between constructs (i.e. self-efficacy, self-regulation, mindfulness) addressed in the modules and symptoms of anxiety and depression, there may also be an improvement in these domains [43–46]. In addition to the weekly modules, participants are asked to complete weekly challenges and keep a web-based daily procrastination diary. This version of StudiCare procrastination was optimized by means of PSD. Principles from all four main categories were included (i.e. primary task support, dialogue support, social support, system credibility), following the definition of the individual principles as described in [28]. Table 1 provides an overview of the specific implementation of principles in this study. StudiCare procrastination and the self-monitoring diary were provided via the secure online platform eSano Research [47].

**Table 1:** Implemented Persuasive System Design features in StudiCare procrastination

Category	Principle	Implementation in StudiCare procrastination
Primary Task Support	Tailoring	IG: opportunity to choose between male or female digital coach Both groups: adaption of modules based on main challenge Optional module about self-esteem
	Self-monitoring	Progress bar in every module Daily diary about procrastination behavior
	Personalization	Opportunity to adjust visual background of program
Dialogue support	Praise	Positive reinforcement from digital coach or eCoach Illustration of confetti after completing a diary entry
	Reminders	Email reminders on module completion Buddy-based reminders on diary completion (*please see cooperation principle*)
	Social Role	IG: female or male digital coach takes a social role by responding to participants’ answers to the tasks
	Similarity	Implementation of case stories
	Liking	Content designed to be visual attractive to the target group of young adults
	Rewards	Collecting the separate pieces of a motivating image which can be seen at the end of each module after each processed task
System credibility	Trustworthiness	Description of empirically supported intervention content and in-depth information about data-security in the introduction module
	Expertise	Expert statements in every module
	Real-world feel	Presentation of developers and team behind StudiCare procrastination
Social Support	Cooperation	Pair of two buddies reminding and supporting each other about daily diary completion (anonymous)
	Social comparison	Information about diary completion of buddy

*Note.* IG = Intervention group

Participants of the IG received the intervention guided by a digital coach (i.e., an avatar). Participants were able to choose between a female or male avatar. The coach provided immediate standardized feedback adapted to the participant’s response for each task. Furthermore, after module completion, participants of the IG received a standardized module summary and message of encouragement. Two business days after module completion, participants received a standardized motivational email. At the end of each module, participants scheduled an appointment for their next module. If participants did not stick to this appointment, they received a reminder email twelve days after the set date by email.

Participants of the CG were guided by a human eCoach, who was a trained psychologist. Prior to the intervention start a training session was held for the eCoaches by the study team on how to create and send feedback. It was also determined how to deal with possible queries from the participants. The eCoaches provided written semi-standardized feedback within two business days after module completion. The feedback was individualized depending on participants’ entries and provided via eSano plattform. The eCoach sent three reminder emails (3, 7, and 10 business days after the module appointment). Participants of the IG and CG received unrestricted access to the health care system (treatment as usual: TAU).

### Outcomes

All participants were invited to complete self-reporting instruments at six measurement time points: baseline (t0), four weeks (t1), eight weeks (t2), twelve weeks (t3), six months (t4), and twelve months (t5) after randomization. Assessments were completed via the secure online survey platform Unipark (www.unipark.com). If participants did not respond to the initial invitation email, they received reminders after three, seven, and ten business days by email. Furthermore, participants who provided a telephone number were contacted by phone after 14 business days.

#### Sociodemographic variables

At baseline, participants were asked about their age, gender, areas of study, nationality, number of study semesters completed, relationship status, current exam preparation, semester break, and experience with psychotherapy.

#### Primary outcome

The primary outcome procrastination was measured using the German version of the Irrational Procrastination Scale (IPS) [36, 48]. The IPS is comprised of nine items presented with a 5-point Likert scale (1 = “*Very seldom or not true to me*” to 5 = “*Very often true or true to me*”). In this study the IPS demonstrated acceptable to excellent internal consistency with McDonald ω between 0.78 (t0) and 0.95 (t1).

#### Secondary outcomes

The Susceptibility to Temptation Scale (STS) measures a further important part of procrastination. The STS consists of eleven items on a 5-point Likert scale (1 = “*Very seldom or not true to me*” to 5 = “*Very often true or true to me*”; 48). The internal consistency of the scale in this study was good to excellent (McDonald ω = 0.89 (t0) − 0.95 (t2/t3)).

The eight-item version of the Patient Health Questionnaire (PHQ-8) [49] was used to assess symptoms of depression. Participants answer on a 4-point Likert scale (0 = “*Not at all*” to 3 = “*Nearly every day*”). In this study, the PHQ-8 displayed good to excellent internal consistency of McDonald ω between 0.86 (t0) and 0.91 (t3).

Symptoms of anxiety were measured by the Generalized Anxiety Disorder Questionnaire [50]. Seven items were assessed on a 4-point Likert scale (0 = “*Not at all*” to 3 = “*Nearly every day*”). McDonald ω in this study was between 0.9 (t0) and 0.93 (t1), pointing to an excellent internal consistency.

The perceived stress severity was assessed using the 4-item version of the Perceived Stress Scale (PSS) [51]. Items were answered on a 5-point Likert scale (0 = “*Never*” to 4 = “*Very often*”). In this study, the PSS showed an internal consistency of McDonald ω between 0.78 (t1) and 0.82 (t2).

The subjective study-related self-efficacy was measured by an area-specific scale (WIRKSTUD), which consists of seven items with a 4-point Likert scale (1 = ”*Not true to me*” to 4 = “*True to me*”) [52]. The internal consistency was good to excellent, as McDonald ω was between 0.85 (t0) and 0.91 (t3).

Social support was measured by the Berliner Social Support Scales (BSSS) using four separate subscales (“perceived social support: emotional”, “perceived social support: instrumental”, “need for social support”, “seeking social support”). In summary, the subscales consist of 17 items, which can be answered on a 4-point Likert scale (1 = ”*Not true to me*” to 4 = “*Totally true to me*”) [53]. Internal consistency was excellent (McDonald ω between 0.93 (t0/1) and 0.95 (t2/3)).

For an additional analysis of the potential mediating effects of shame and self-esteem on procrastination, we added three further questionnaires: the German self-esteem scale (RSES), fear of negative evaluation scale (SANB-5), and an ad hoc item assessing feelings of shame. The results of this secondary analysis will be presented in another publication.

#### Adherence

Intervention’s adherence was defined as completing more than 80% of the main intervention modules [54].

#### Working alliance

Therapeutic alliance is one of the key mechanisms of change in psychotherapy [55, 56]. We assessed participants’ alliance to the intervention utilizing the German version of the Working Alliance Inventory for guided Internet interventions (WAI-I) [57]. Twelve items were answered on a 5-point Likert scale (1 =” *seldom*” to 5 = “*always*”). Internal consistency was good to excellent with McDonald ω between 0.81 (t3) and 0.96 (t2).

#### Subjective negative side effects

Subjective negative side effects were measured with the Negative Effects Questionnaire (NEQ). The original version of the NEQ consists of 20 items [58]. Given the health promotion focus of our study we removed item 10, which assesses suicidal ideation. Each item asks whether a specific side effect occurred. If yes, participants were asked whether they relate this side effect to their intervention participation and how strongly they were impacted by the side effect (4-point Likert scale 1 = “*not at all*” to 4 = “*very strong*”). Internal consistency was acceptable (McDonald ω = 0.81).

#### Feasibility

To gain insights into the perceived usability of the IMI, the short version of the User Experience Questionnaire (UEQ-S) was implemented after the second and fifth modules. It consists of eight items ranging from − 3 (= *fully agree with negative term*) to 3 (= *fully agree with positive term*) targeting the dimensions aesthetics, pragmatic quality, and hedonic quality. Thus, values < -0.8 indicate a negative, -0.8 to 0.8 neutral, and > 0.8 a positive user experience. Subscales’ reliability are excellent (hedonic quality: McDonald ω = 0.94) or good (pragmatic quality: McDonald ω = 0.85) [59].

After the fifth module, IG participants were invited to provide qualitative feedback. First, they were asked to rate how much they enjoyed the interaction with the digital coach on a 7-point Likert scale ranging from 1 (= *not at all*) to 7 (= *very much*). Second, an open-ended question was provided to gather feedback on the perceived guidance.

### Statistical analysis

All analyses were performed with the software R [60]. A two-sided significance level of p < .05 was applied. For all outcomes at baseline, the mean and standard deviation were reported. Internal consistency of outcome measurements was explored using McDonald ω [61]. Differences in adherence were assessed using Welch two sample t-test.

Analyses were based on intention to treat (ITT). We assumed missing data to be missing at random and handled it by Full-Information Maximum Likelihood (FIML) estimation [62]. Maximum Likelihood Robust (MLR) estimators were used [63]. Changes in continuous outcomes, group differences, and potential sensitivity analysis of potential predictors were analyzed using latent growth curve models based on structural equation modeling (SEM). SEM were extended with mean structure by using effects coding and the requirement of measurement invariance was tested [64, 65]. Since χ2-tests are too sensitive to evaluate the absolute model fit for each applied model here [66, 67], the fit indices Comparative Fit Index (CFI) [68], the robust Root Mean Square Error of Approximation (RMSEA) [69], and the Standardized Root Mean Square Residual (SRMR) [70] were taken into consideration [71]. We applied standard modeling criteria as cut-off values for an acceptable goodness of fit: CFI > 0.95; RMSEA < 0.06; SRMR < 0.08. For the analysis of the latent growth curve model we used the R-package lavaan [72]. With regard to group differences, the outcomes of the latent growth curve model were supported by Cohen’s *d*. These effect sizes were based on data imputed by Multivariate Imputation by Chained Equations (MICE) [73]. We report on the slope (γ), the p-value, and the Cohen’s *d* with the corresponding 95%-Confidence Interval (95%-CI) for each effectiveness outcome and related predictors. Negative slopes indicate improvement. Regarding group effects, a non-significant value points to no statistically relevant influence of the group allocation. Negative Cohen’s *d* favors IG unless it is indicated that higher values indicate a better outcome.

Qualitative feedback on user experience in IG was analyzed with content analysis on an observed data level [74]. In a first step, the feedback was categorized in positive or negative evaluation. Second the feedback was categorized by different coach characteristics (usefulness, conversation, interaction, visual design).

## Results

**Fig. 1:**
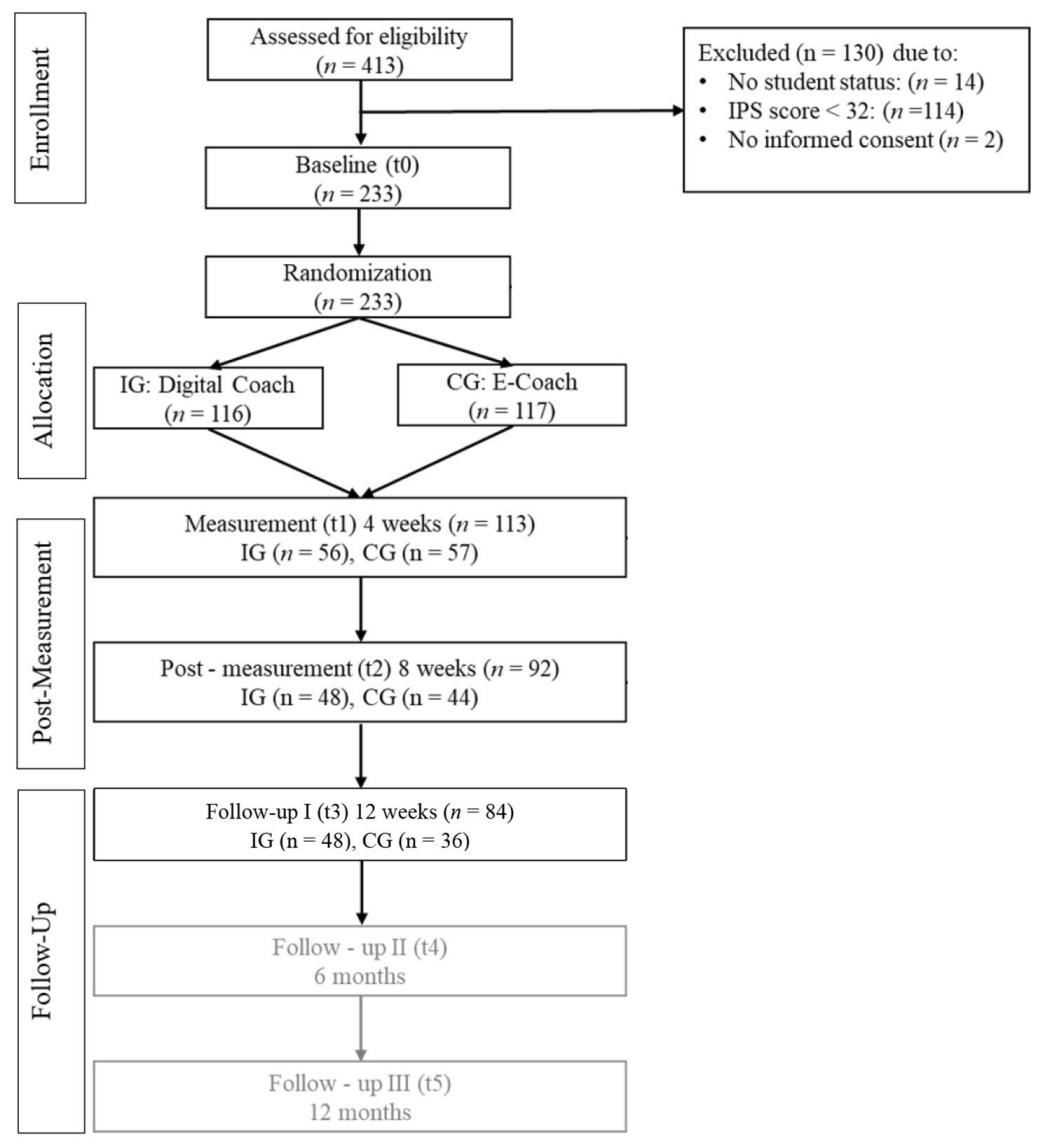
Study Flowchart. *Note.* The results Follow-Up II and III are not within the scope of this publication. IG = Intervention group; CG = Control group

A total of 233 participants were randomized either to StudiCare procrastination guided by a digital coach (IG) or to StudiCare procrastination guided by a human eCoach. For further information on the study dropout please see the study flow (Fig. 1). There was no significant difference regarding baseline IPS scores (t1: *p* = .72, t2: *p* = .75, t3: *p* = .31) between participants who dropped out and those who did not. The same applies to gender (t1: *p* = .25, t2: *p* = .62, t3: *p* = .07) and age (t1: *p* = .49, t2: *p* = .14, t3: *p* = .95). IG and CG participants’ baseline characteristics can be found in Table 2. Procrastination was not significantly associated with either being in a semester break (*t*(155) = 0.46, *p* = .65) nor preparing for an exam (*t*(185) = − 0.39, *p* = .70).

**Table 2:** Baseline Characteristics across and per group

	All participants (N = 233) % (n)	IG (*n* = 116) % (n)	CG (*n* = 117) % (n)
Age *M* (*SD*)	26.21 (5.3)	26.16 (5.53)	26.26 (5.18)
Gender, female Diverse	60 (140), 1 (3)	55 (64), 2 (2)	65 (76), 1 (1)
**Citizenship**			
Germany	92 (215)	95 (110)	90 (105)
Switzerland	1 (2)	1 (1)	1 (1)
EEA member	3 (8)	3 (3)	4 (5)
No EEA member	3 (6)	1 (1)	4 (5)
No information	0.005 (1)	0	1 (1)
**Study characteristics**			
Semester break	32 (75)	29 (34)	35 (41)
Exam phase	64 (148)	65 (75)	62 (73)
Study semesters *M* (*SD*)	10.75 (6.73)	15.55 (6.25)	10.96 (7.20)
**Field of study**			
Biology/ Chemistry	5 (12)	6 (7)	4 (5)
Business and Law	12 (27)	15 (17)	8 (10)
Educational Sciences	15 (35)	13 (15)	17 (20)
Engineering	14 (32)	13 (15)	15 (17)
Informatics	11 (26)	13 (15)	9 (11)
Linguistics, Culture, and Geography	14 (33)	15 (17)	14 (16)
Mathematics and Physics	7 (16)	6 (7)	8 (9)
Medicine and Pharmaceutics	8 (18)	6 (7)	9 (11)
Other sciences	4 (9)	4 (5)	3 (4)
No information	1 (3)	2 (2)	1 (1)
**Psychotherapy experience**			
Currently	13 (31)	12 (14)	15 (17)
longer than 3 months ago	24 (55)	21 (24)	26 (31)
Waiting list	3 (8)	2 (2)	5 (6)
No Experience	60 (139)	66 (76)	54 (63)
**Treatment for procrastination**			
Internet	27 (62)	31 (36)	22 (26)
Consultation	8 (18)	3 (6)	10 (12)
Family doctor	2 (4)	1 (1)	3 (3)
Psychotherapist	11 (26)	11 (13)	11 (13)
Psychiatrist	2 (4)	1 (1)	3 (3)
Other	4 (9)	4 (5)	3 (4)
**Mental health outcomes**			
**IPS***M* (*SD*)	37.43 (3.59)	37.16 (3.81)	37.70 (3.36)
**STS***M* (*SD*)	42.01 (6.60)	41.79 (6.89)	42.23 (6.32)
**WIRKSTUD***M* (*SD*)	17.01 (3.98)	17.14 (4.08)	16.89 (3.88)
**PHQ-8*** M* (*SD*)	11.55 (5.02)	11.40 (5.17)	11.69 (4.88)
**GAD-7*** M* (*SD*)	9.39 (4.91)	9.33 (5.29)	9.37 (4.53)
**PSS***M* (*SD*)	9.20 (2.90)	8.82 (3.05)	9.58 (2.70)
**BSSS***M* (*SD*)	50.07 (8.65)	51.03 (8.26)	49.11 (8.96)
emotional *M* (*SD*)	13.41 (2.59)	13.61 (2.55)	13.21 (2.63)
instrumental *M* (*SD*)	12.8 (2.73)	13.00 (2.65)	12.60 (2.80)
need for support *M* (*SD*)	11.3 (2.62)	11.30 (2.45)	11.29 (2.79)
support seeking *M* (*SD*)	12.57 (3.17)	13.12 (2.94)	12.02 (3.11)

*Note.* IPS = Irrational Procrastination Scale, STS = Susceptibility to Temptation Scale, WIRKSTUD = Study-related self-efficacy, PHQ-8 = 8 item version of the Patient Health Questionnaire, GAD-7 = 7 item version of the General Anxiety Disorder questionnaire, PSS = Perceived Stress Scale, BSSS = Berliner Social Support Scale, M = Mean, SD = Standard deviation, IG = Intervention group, CG = Control group

### Primary outcome

The IPS showed scalar invariance in the presence of partial metric invariance (additional file 1). Both groups demonstrated a significant reduction of latent IPS scores across time points (γ = -0.79; *p* < .001). The calculated Cohen’s *d* indicate a medium to large effect size (t1: Cohen’s *d* = − 0.43, 95%-confidence interval (CI) [-0.61; -0.25]; t2: Cohen’s *d* = − 0.72, 95%-CI [-0.91; -0.53]; t3: Cohen’s *d* = − 0.89, 95%-CI [-1.08; -0.7]). There was no significant time x group interaction regarding IPS (γ = -0.03, *p* = .84), indicating that group had no significant effect on IPS improvement over time. The effect sizes of group differences support this finding. For each measurement point, levels did not exceed the non-inferiority margin of Cohen’s *d* = − 0.3 (t1: Cohen’s *d* = 0.04, 95%-CI [-0.22; 0.30]; t2: Cohen’s *d* = -0.03, 95%-CI [-0.29; 0.23]; t3: Cohen’s *d* = 0.08, 95%-CI [-0.18; 0.34]).

When it comes to potential predictors of the IPS improvement, number of modules (γ= − 0.20, *p* = .25), number of completed study semesters (γ = 0.11, *p* = .40), and gender (γ = − 0.14, *p* = .43) had no significant predicting influence on the time effect across groups.

### Secondary outcomes

Regarding measurement invariance, we found scalar invariance for the following outcomes: PHQ-8, GAD-7, PSS, and partial scalar invariance for the STS. The outcome WIRKSTUD showed partial metric and partial scalar invariance. The fit indices for all applied models can be found in additional file 1. Results on the STS showed a significant reduction of susceptibility to temptations across both groups (γ = − 0.62, *p* < .001) with medium effect sizes (Cohen’s *d* = -0.85 to -0.41). There were also significant reductions across groups in depression scores (γ = -0.15, *p* < .001), anxiety scores (γ = -0.25, *p* = .01), perceived stress (γ = -0.36, *p* < .001) with small to medium effect sizes (Cohen’s *d* = -0.73 to -0.23). Regarding study related self-efficacy, we found a significant improvement across groups (γ = 0.38, *p* < .001) with medium effect sizes (Cohen’s *d* = 0.40 to 0.79).

When it comes to potential non-inferiority of the IG compared to the CG, the group allocation had no significant influence on the improvement of the susceptibility to temptations (STS: γ = -0.01, *p* = .93), of the depression scores (PHQ: γ = 0.26, *p* = .09), of the anxiety scores (GAD: γ = 0.25, *p* = .09) or of the self-efficacy (WIRKSTUD: γ = -0.20, *p* = .10). Between-group differences showed small to medium effect sizes (Cohen’s *d* between − 0.02 and 0.52). However, group allocation had a significant influence on the reduction of perceived stress (PSS: γ = 0.26, *p* = .04) with small effect sizes (across time points: Cohen’s *d* = -0.04 to 0.26), favoring the CG.

In terms of potential predicting variables of the time effects across groups, we found that neither gender, nor the number of modules or semesters studied predicted any effect. Detailed results of all outcomes and the predictors are provided in Table 3.

**Table 3:** Results on primary and secondary outcomes

	Time effects across both groups			Time x group interaction effects			Time x predictor effects	
**Outcome**	γ	*p*	Cohen’s *d* [95%-CI]	γ	*p*	Cohen’s *d* [95%-CI]	γ	*p*
IPS	-0.79	< 0.001	t1: − 0.43 [-0.61; -0.25] t2: − 0.72 [-0.91; -0.53] t3: − 0.89 [-1.08; -0.70]	-0.03	0.84	t1: 0.04 [-0.22; 0.30] t2: -0.03 [-0.29; 0.23] t3: 0.08 [-0.18; 0.34]	Gender: − 0.14 Modules: − 0.2 Study semester: 0.11	0.43 0.25 0.40
STS	-0.62	< 0.001	t1: − 0.41 [-0.59; -0.23] t2: − 0.51 [-0.7; -0.34] t3: − 0.85 [-1.04; -0.66]	-0.04	0.75	t1: -0.26 [-0.52; -0.00] t2: -0.11 [-0.37; 0.15] t3: -0.23 [-0.49; 0.03]	Gender: -0.02 Modules: − 0.06 Study semester: 0.13	0.9 0.75 0.39
PHQ	-0.15	< 0.001	t1: − 0.30 [-0.48; -0.12] t2: − 0.41 [-0.59; -0.23] t3: − 0.50 [-0.68; -0.32]	0.26	0.09	t1: -0.15 [-0.41; 0.11] t2: 0.21 [-0.05; 0.47] t3: -0.09 [-0.35; 0.17]	Gender: − 0.13 Modules: 0.16 Study semester: 0.10	0.6 0.45 0.64
GAD	-0.25	0.01	t1: − 0.23 [-0.41; -0.05] t2: − 0.37 [-0.55; -0.19] t3: − 0.38 [-0.56; -0.20]	0.25	0.09	t1: -0.29 [-0.55; -0.03] t2: 0.51 [0.25; 0.77] t3: 0.37 [0.11; 0.63]	Gender: 0.07 Modules: 0.1 Study semester: 0.17	0.58 0.5 0.15
PSS	-0.36	< 0.001	t1: − 0.44 [-0.62; -0.26] t2: − 0.60 [-0.79; -0.41] t3: − 0.73 [-0.92; -0.54]	0.26	0.04	t1: -0.12 [-0.38; 0.14] t2: 0.26 [0.00; 0.52] t3: -0.04 [-0.30; 0.22]	Gender: − 0.06 Modules: 0.02 Study semester: 0.15	0.62 0.88 0.12
WIRKSTUD*	0.38	< 0.001	t1: 0.40 [0.21; 0.58] t2: 0.60 [0.41; 0.79] t3: 0.79 [0.6; 0.98]	-0.20	0.10	t1: 0.18 [-0.08; 0.44] t2: -0.02 [-0.28; 0.24] t3: -0.52 [-0.78; -0.26]	Gender: − 0.13 Modules: 0.08 Study semester: − 0.07	0.37 0.56 0.48
BSSS perceived, emotional support*	-0.49	< 0.001	t1: 0.03 [-0.15; 0.21] t2: 0.18 [-0.00; 0.36] t3: − 0.11 [-0.29; 0.07]	-0.19	0.22	t1: 0.28 [0.02; 0.54] t2: 0.17 [-0.08; 0.43] t3: -0.05 [-0.31; 0.21]	Gender: − 0.17 Modules: − 0.02 Study semester: − 0.33	0.75 0.89 0.02
BSSS perceived, instrumental support*	-0.48	< 0.001	t1: 0.12 [-0.06; 0.3] t2: 0.28 [0.10; 0.46] t3: 0.05 [-0.13; 0.23]	-0.16	0.46	t1: 0.60 [0.34; 0.86] t2: 0.22 [-0.04; 0.48] t3: -0.03 [-0.29; 0.23]	Gender: 0.26 Modules: − 0.06 Study semester: − 0.29	0.23 0.77 0.08
BSSS Need for support*	-0.43	< 0.001	t1: − 0.12 [-0.3; 0.06] t2: − 0.14 [-0.32; 0.04] t3: − 0.48 [-0.66; -0.3]	-0.08	0.58	t1: 0.11 [-0.15; 0.37] t2: -0.09 [-0.35; 0.17] t3: -0.08 [-0.34; 0.18]	Gender: − 0.03 Modules: 0.29 Study semester: 0.07	0.85 0.07 0.71
BSSS Support Seeking*	-0.40	< 0.001	t1: − 0.12 [-0.3; 0.06] t2: 0.13 [-0.05; 0.31] t3: 0.0 [-0.18; 0.18]	-0.47	0.01	t1: 0.04 [-0.22; 0.30] t2: -0.02 [-0.28; 0.24] t3: -0.18 [-0.44; 0.08]	Gender: 0.23 Modules: − 0.18 Study semester: − 0.36	0.29 0.39 0.04

*Note.* *higher scores indicate better outcome; CI = Confidence Interval;

IPS = Irrational Procrastination Scale, STS = Susceptibility to Temptation Scale, WIRKSTUD = Study-related self-efficacy, PHQ-8 = 8 item version of the Patient Health Questionnaire, GAD-7 = 7 item version of the General Anxiety Disorder questionnaire,

PSS = Perceived Stress Scale, BSSS = Berliner Social Support Scale

### Adverse events

In the IG, 199 adverse events were reported in the NEQ, of which 85 were associated with the intervention. In the CG, 194 adverse events, 85 related to intervention participation, were reported. There were no significant differences between the IG and the CG in the frequency of reported adverse events. The impact of the adverse events was rated between *M* = 1.33 and *M* = 3.60 in the IG and *M* = 1.0 and *M* = 4.0 in the CG. Please see Table 4 for detailed information.

**Table 4:** Negative effects of intervention participation for both groups

Negative effect	Number of impacted participants n (%)		*p*	Impact of negative effect *M* (*SD*)		Negative impact from treatment n (%)	
	CG	IG		CG	IG	CG	IG
Problems with sleep	16 (14)	14 (12)	0.51	3.19 (0.98)	3.0 (0.96)	0	0
More stress	18 (15)	18 (16)	0.83	3.28 (1.13)	3.28 (0.83)	3 (17)	5 (28)
More anxiety	6 (5)	7 (6)	1.0	4.00 (0.89)	3.0 (0.58)	0	3 (43)
More worried	12 (10)	10 (9)	0.63	3.08 (0.67)	2.70 (0.68)	1 (8)	3 (30)
More hopelessness	11 (9)	7 (6)	0.29	3.64 (1.03)	3.43 (0.98)	2 (18)	2 (29)
More unpleasant feelings	16 (13)	17 (15)	1.0	3.13 (0.89)	2.71 (1.21)	7 (44)	8 (47)
Issue got worse	8 (7)	11 (10)	0.62	2.75 (1.34)	3.27 (1.68)	1 (13)	3 (27)
Resurface of unplesant memories	16 (14)	16 (14)	0.83	2.75 (0.93)	2.75 (1.13)	8 (50)	5 (31)
Fear other people find out about treatment	6 (5)	5 (4)	0.75	2.00 (1.90)	2.60 (0.55)	3 (50)	0
Feeling ashamed	5 (4)	3 (3)	0.47	1.40 (1.14)	1.33 (1.16)	4 (80)	1 (33)
Stop thinking things could get better	12 (10)	8 (7)	0.31	2.83 (1.03)	3.00 (1.07)	4 (33)	4 (50)
Start thinking issue could not be made any better	13 (11)	18 (16)	0.51	3.46 (1.27)	2.94 (1.16)	6 (46)	11 (61)
Developed dependency on treatment	1 (1)	1 (1)	1.0	1.0	2.0	1 (100)	1 (100)
Not always understand treatment	7 (6)	7 (6)	1.0	2.14 (0.69)	2.57 (1.27)	6 (86)	6 (86)
Not always understand therapist	2 (2)	3 (3)	1.0	3.5 (0.71)	2.0 (1.0)	2 (100)	3 (100)
No confidence in treatment	10 (9)	9 (8)	0.80	2.40 (0.70)	2.44 (1.51)	9 (90)	7 (78)
No results produced by treatment	17 (15)	26 (22)	0.15	2.76 (1.15)	2.62 (1.36)	12 (71)	17 (65)
Expectations for treatment not fulfilled	13 (11)	14 (12)	1.0	2.46 (1.20)	2.71 (1.38)	13 (100)	12 (86)
Feeling of treatment as not motivating	5 (4)	5 (4)	1.0	2.40 (1.14)	3.60 (1.14)	3 (60)	4 (80)

*Note.* Measured by the Negative Effects Questionnaire (NEQ), 19 item version; M = Mean, SD = Standard deviation, IG = Intervention group (*n* = 48), CG = Control group (*n* = 44)

### Adherence

In the IG, 84% of participants completed the introduction, 72% the first module, 53% the second, 41% the third, 34% the fourth, and 24% the fifth module. 14% completed the optional module. On average, participants of the IG completed *M* = 2.83 (*SD* = 2.11) modules. Overall, the adherence rate in the IG was 34%. In the CG, 88% completed the introduction, 80% the first module, 62% the second, 50% the third, 36% the fourth, and 31% the fifth module. 20% completed the optional module. This points to an adherence rate of 36% in the CG, with an average of *M* = 3.17 (*SD* = 2.08) of completed modules. Regarding adherence rate, group differences were ≤ 9% with highest (9%) in modules two and three. In both groups, the highest attrition occurred after the first module. There was no significant difference in adherence between IG and CG (*t*(198) = 1.15, *p* = .25).

The number of completed modules was not significantly predicted by being in exam phase (*β* = 0.57, *p* = .06) or having semester break (*β* = -0.04, *p* = .90).

In total, 172 participants used the buddy reminder at least once. On average, participants sent 3.47 (*SD* = 5.29) reminders. 83 (72%) participants of the IG (*M* = 3.06, *SD* = 4.24) and 89 (76%) participants of the CG (*M* = 3.84, *SD* = 6.12) used the buddy feature (Cohen’s *d* = 0.15, *p* = .33).

### Therapeutic alliance

The observed rated therapeutic alliance for both groups and across groups can be found in Table 5. On an ITT-basis, group has no significant influence on the slope (γ = 0.07, *p* = .61) or the intercept (γ = − 0.10, *p* = .31) in the overall rating. This means that there is no significant difference in the therapeutic alliance between the groups, either at baseline or over time.

**Table 5:** Observer-based rating of therapeutic alliance

	4 weeks (t1)			8 weeks (t2)			12 weeks (t3)		
	IG *n* = 56	CG *n* = 57	Both	IG *n* = 48	CG *n* = 44	Both	IG *n* = 48	CG *n* = 36	Both
Overall *M* (*SD*)	2.71 (0.82)	2.86 (0.74)	2.79 (0.78)	2.75 (0.93)	3.17 (0.79)	2.96 (0.88)	2.85 (0.95)	3.11 (0.84)	2.96 (0.91)
Task and goal agreement *M* (*SD*)	2.92 (0.90)	2.92 (0.78)	2.92 (0.84)	2.99 (0.97)	3.22 (0.81)	3.10 (0.90)	3.09 (0.93)	3.14 (0.81)	3.11 (0.88)
Bond *M* (*SD*)	2.29 (1.10)	2.74 (1.03)	2.52 (1.08)	2.29 (1.04)	3.09 (0.98)	2.67 (1.09)	2.38 (1.26)	3.06 (1.22)	2.67 (1.28)

Note. IG = intervention group; CG = control group; M = mean; SD = standard deviation

When it comes to the subscale bond there is a significant difference on the intercept (t1) between groups (γ = -0.26, *p* = .02) but no significant influence of group on the slope (γ = -0.19, *p* = .38). Concerning the subscale goal and task there is neither a significant influence of the group on the intercept (γ = − 0.07, *p* = .50) nor on the slope (γ = 0.15, *p* = .33).

### Feasibility

After the second module, 73 participants of the CG rated the user experience with *M* = 0.57 (*SD* = 1.11) and 61 participants of the IG with *M* = 0.95 (*SD* = 1.23), indicating neutral and good user experience, respectively. After the fifth module, 36 participants of the CG rated the user experience with *M* = 0.99 (*SD* = 1.00) and 27 participants of the IG with *M* = 0.83 (*SD* = 0.97), suggesting a good user experience in both groups.

### Qualitative user feedback

The digital coach was rated with *M* = 3.89 (*SD* = 1.42) by 27 participants of the IG after the fifth module. Four participants described the digital coach as useless, whereas four mentioned its helpfulness. Nine participants felt the conversation was impersonal and six participants felt it was motivating. Four participants missed interaction with the coach, whereas two mentioned the positivity of instant answers of the digital coach. Two persons would like the design of the coach to be more contemporary.

## Discussion

This non-inferiority study showed that the guidance by a digital coach does not exceed a non-inferiority margin of − 0.3 in comparison to human-based guidance in an iCBT targeting academic procrastination. This result contradicts previous studies, which indicate that automated support in IMI is effective but inferior to human support [30]. However, the latest studies suggest that IMI including elements designed to increase engagement (“second-generation interventions”) as is the case with PSD strategies, may produce effects comparable to clinician-guided treatments [33, 75, 76]. The result of the non-inferiority found in our study support that assumption and indicate that guidance from a digital coach is an effective alternative for students with procrastination that saves resources.

Regarding adherence, there was no significant difference between the two intervention groups, which is contradictory to the finding of a currently published meta-analysis. Musiat et al. (2022) find that on average, the completion rate in human-guided IMI targeting mental health is 12% higher than in unguided interventions [77]. The PSD optimization could be a possible explanation for the non-inferiority finding in our study. In general, despite the engagement-promoting PSD principles, we found a comparably low adherence rate of 34% (IG) and 36% (CG) in this study compared with Schmidt et al. [78], who reported an average dropout of 32% from iCBT programs for depression. Several reasons for that finding can be discussed. First, the timing of the iCBT during the semester could have played a decisive role. We found that the adherence tend to be higher in participants preparing for exams. Notably, this finding just misses the significance level. However, it is possible that students are most aware of the consequences of procrastination during this time, thus are more motivated. This finding would suggest just-in-time adaptive interventions to be a promising further development [79], that offer a procrastination intervention during exam preparations. Furthermore, a large number of participants had the feeling that they did not benefit from the intervention, what might be a further reason for low adherence [80]. Regarding adherence in the IG, the optimization of the digital coach might be valuable. Although, the WAI-I showed non-inferiority of IG to CG in the overall therapeutic alliance, in the subscale bond there was inferiority. So, participants of the IG experienced lesser feelings that the digital coach likes them, respects them, appreciates them, and is interested in their well-being. Zalaznik et al. [81] indicate that the participants’ connection to the IMI, which is measured by the other subscales of the WAI-I, predicts symptom outcomes, whereas the relationship with the coach (subscale bond) is important for adherence. Complementary, the qualitative feedback provided by IG participants on the digital coach revealed a need for optimization; this might further improve user experience, which is currently rated as neutral-positive by the UEQ-S, and thus increase adherence in the IG [59]. First, the digital coach could be more individualized by the implementation of a chatbot system [82]. Previous studies indicate the acceptance and usefulness of chatbots in IMI for mental health [e.g., 83]. Second, the engagement with the digital coach may be improved by applying principles of gamification. Principles such as challenges or storytelling have demonstrated the ability to increase the passion and emotional involvement of participants [84]. Including principles of gamification may also be a strategy to strengthen the use of the buddy-based diary. A challenge between the buddies or being able to reach common milestones could be motivating [85].

Finally, however, the relevance of the adherence of this target group must be questioned. So, our sensitivity analysis showed that the number of completed modules did not predict effectiveness in this trial, which is contradictory to previous studies on IMI[e.g., 86], but in line with the findings of a meta-analysis about face to face interventions targeting procrastination [17]. In that meta-analysis intervention duration had no significant moderating effect on symptom improvement [17]. This result raises the question of whether all five modules of StudiCare procrastination are needed to exploit its full potential. A smaller number of modules may be sufficient to have a positive effect on procrastination. Research on CBT shows that symptom improvement can occur before the introduction of formal treatment elements (e.g., cognitive restructuring). It is hypothesized that non-specific treatment factors decrease feelings of hopelessness in participants at the beginning of treatment and catalyze symptom improvement [87]. Adherence may not be of high importance in the target group of students with procrastination. Of course, it remains unclear whether low adherence has any influence on the long-term effects. Further analysis of the follow-up data is necessary.

In both groups, self-reported procrastination in students was reduced with a medium effect size, measured with the IPS and STS across groups. The finding that IMI can reduce procrastination in college students is in line with previous studies which also reported medium within-group effect sizes for guided and unguided groups [21, 88]. When it comes to potential predictors of the improvement, neither study semester nor gender had an impact on improvements of the different outcomes across groups. This indicates that the intervention may work independently of certain baseline characteristics.

In general, it is noticeable that the target group appears to be under significant psychological strain. This is shown, among other things, by the high anxiety and depression scores, which on average are above the generally applicable cut-off scores for the PHQ-8 and GAD-7 [89, 90]. The significant correlation between depression and procrastination has been observed in numerous studies [14, 91]. Encouragingly, participation in the intervention may significantly reduce depression. The effects are small to medium and of clinical relevance [92]. Thereby, at no measurement time point did the IG show inferiority to the CG of clinically relevant effect size.

Regarding exploratory analyses on further secondary outcomes, no clear pattern across the different outcome variables can be found at which point human guidance becomes superior. For example, there was IG inferiority for anxiety from measurement point t2. It is possible that it depends on the outcome measure whether human guidance is necessary. At the same time, it is important to observe the follow-up surveys to discover possible patterns. In general, students with procrastination could be a suitable target group for IMI with digital guidance [88, 93].

### Limitations

Besides some strength of the study as the applied statistical model which has several benefits over traditional analysis methods for longitudinal data and has been proven to have a higher level of statistical power [94], there are some limitations that must be considered when interpreting the findings. First, there is a high study dropout of 64%. To prevent bias, the data were analyzed based on ITT. FIML and MICE are considered an adequate method to deal with missing data [62, 73]. Nevertheless, causes of dropout should also be explored. On one hand, participants did not receive any incentives, which are considered effective in preventing study dropout. Furthermore, the participants had to answer several questionnaires at five measurement points. The time constraint may have been considered too large. In addition to the standardized reminder strategy, individualization of reminders could be helpful [95]. It should be noted that the dropout rate is higher than for other IMI among college students, e.g., for mindfulness [96, 97] or social anxiety [98]. This could provide evidence for a target group-specific influence. However, in our study the baseline procrastination score did not have a significant influence on study dropout. Nevertheless, in qualitative research a lack of motivation in the target group or an inability to keep up with the treatment schedule are discussed. The authors conclude that especially in IMI for procrastination a shortening of modules can be helpful [99]. This might also be transferable to the length of the questionnaires, which may have been too many and frequently delivered.

Second, there were some technical issues at the beginning of the study. During the first weeks, there were some difficulties in accessing the program’s content. The participants were immediately informed about this. However, it cannot be ruled out that these technical problems impacted the perceived user experience of the program.

Third, outcomes were only assessed through self-report, which may lead to potential sampling bias [100]. Diagnostic interviews as external assessment, could help to validate self-reported data.

Fourth, secondary outcome analyses were explorative and might be underpowered. They should be interpreted with caution.

Fifth, extensive research and considerations were made for the definition of the non-inferiority margin. Nevertheless, some uncertainty remains, as there are no generally valid figures for a clinically relevant effect in this target group to date.

## Conclusion and further directions

The Persuasive Design optimized StudiCare procrastination iCBT is effective in reducing academic procrastination in college students and correlated symptoms such as depression. Guidance by a digital coach is not inferior to human guidance. These results indicate that an IMI guided by a digital coach is a low-threshold treatment alternative for students affected by procrastination. The analysis of the follow-up data will show whether this effect lasts in the long-term. This study contributes to the growing evidence of the automation of iCBT. Digital guidance further facilitates the scalability and implementation of such interventions and produces less costs for guidance.

### Electronic supplementary material

Below is the link to the electronic supplementary material.

Supplementary Material 1

## Data Availability

The datasets used and/or analyzed during the current study are available from the corresponding author on reasonable request.

## Abbreviations

BSSS: Berliner Social Support Scale
CG: Control group
GAD-7: 7 item version of the General Anxiety Disorder questionnaire
iCBT: Internet- and mobile-based cognitive behavioral therapy
IMI: Internet- and mobile-based intervention
IG: Intervention group
IPS: Irrational Procrastination Scale
M: Mean
PHQ-8: 8 Item version of the Patient Health Questionnaire
PSD: Persuasive system design
PSS: Perceived Stress Scale
SD: Standard deviation
STS: Susceptibility to Temptation Scale
WIRKSTUD: Study-related self-efficacy
95%-CI: 95% Confidence Interval
