# Supplementary material for: StudiCare procrastination - Randomized controlled non-inferiority trial of a persuasive design-optimized internet- and mobile-based intervention with digital coach targeting procrastination in college students
Source: BMC Psychol. 2023 Sep 12;11:273. doi: 10.1186/s40359-023-01312-1 (PMC10496391; doi:10.1186/s40359-023-01312-1)
Supplement: Supplementary file 1 — Supplementary Material 1 [file 40359_2023_1312_MOESM1_ESM.docx]

### *Additional file 1*. Fit indices for each outcome

| Outcome | CFI | RMSEA | SRMR |
| --- | --- | --- | --- |
| IPS | 0.90 | 0.04 | 0.08 |
| STS | 0.86 | 0.05 | 0.09 |
| PHQ-8 | 0.90 | 0.05 | 0.08 |
| GAD | 0.95 | 0.04 | 0.08 |
| PSS | 0.97 | 0.03 | 0.07 |
| WIRKSTUD | 0.93 | 0.04 | 0.09 |
| BSSS perceived emotional support | 0.99 | 0.03 | 0.06 |
| BSSS perceived instrumental support | 0.97 | 0.05 | 0.07 |
| BSSS need for support | 0.93 | 0.06 | 0.08 |
| BSSS support seeking | 0.93 | 0.05 | 0.08 |

*Note.* For each outcome variable the table presents the fit indices for the model for symptom change across groups in the first line. In the second line the fit indices for the between group model are presented. CFI = Comparative Fit Index; RMSEA = Root Mean Square Error of Approximation; SRMR = Standardized Root Mean Square Residual; IPS = Irrational Procrastination Scale, STS = Susceptibility to Temptation Scale, WIRKSTUD = study-related self-efficacy, PHQ-8 = 8 item version of the Patient Health Questionnaire, GAD-7 = 7 item version of the General Anxiety Disorder questionnaire, PSS = Perceived Stress Scale, BSSS = Berliner Social Support Scale
